# Role of Intracoronary Imaging in Myocardial Infarction with Non-Obstructive Coronary Disease (MINOCA): A Review

**DOI:** 10.3390/jcm12062129

**Published:** 2023-03-08

**Authors:** Irene Borzillo, Ovidio De Filippo, Rossella Manai, Francesco Bruno, Emanuele Ravetti, Alma Andrea Galanti, Rocco Vergallo, Italo Porto, Gaetano Maria De Ferrari, Fabrizio D’Ascenzo

**Affiliations:** 1 Division of Cardiology, Cardiovascular and Thoracic Department, “Città della Salute e della Scienza” Hospital, 10126 Turin, Italy; 2 Department of Medical Sciences, University of Turin, 10126 Turin, Italy; 3 Department of Internal Medicine, University of Genoa, 16132 Genoa, Italy; 4 Cardiology Unit, IRCCS Ospedale Policlinico San Martino, 16132 Genoa, Italy

**Keywords:** intracoronary imaging, intravascular ultrasounds, optical coherence tomography, myocardial infarction, cardiac magnetic resonance imaging

## Abstract

Myocardial infarction with non-obstructive coronary artery disease occurs in 6% to 15% of all presentation of myocardial infarctions. The pathophysiologic mechanisms of MINOCA include epicardial vasospasm, coronary microvascular disorder, spontaneous coronary artery dissection, and coronary thrombus/embolism. The diagnosis is challenging, supported by intracoronary imaging with intravascular ultrasound (IVUS) and optical coherent tomography (OCT), coronary physiology testing, and cardiac magnetic resonance imaging (CMR). OCT is able to identify atherosclerotic causes of MINOCA (plaque erosion, plaque rupture, and calcified nodule) and nonatherosclerotic causes (spontaneous artery dissection, and spasm). In this review, we summarize the performance of the two intracoronary imaging modalities (IVUS and OCT) in MINOCA and discuss the importance of supplementing these modalities with CMR in order to drive target therapy.

## 1. Introduction

Myocardial infarction with nonobstructive coronary arteries (MINOCA) is a relatively common clinical entity characterized by evidence of myocardial injury in the absence of obstructive (>50%) coronary stenoses on angiography and in the absence of an alternative diagnosis for the acute presentation (i.e., sepsis, pulmonary embolism, etc.) [1]. MINOCA accounts for 6% to 15% of patients with spontaneous myocardial infarction (MI) and disproportionately affects women [2]. In MINOCA patients, anxiety and depression are more frequent compared to patients with obstructive coronary artery disease [3]. There is a difference also in the period of the year when MINOCA occurs. The incidence of MINOCA increases in summer and autumn [4].

The cardiovascular risk factor profile of MINOCA patients does not substantially differ from the population with obstructive myocardial infarction (MI-CAD), except for a lower prevalence of hyperlidipemia [5]. At the time of diagnosis, about two-thirds of MINOCA patients have an electrocardiographic pattern classifiable as MI in the absence of ST-segment elevation MI (NSTEMI), while in one-third of the cases, the presentation is ST-segment elevation myocardial infarction (STEMI) [5].

Although the underlying cause of MINOCA often remains undetermined, the overall prognosis may be adverse, with a 1-year mortality of approximately 4.7% [5]. The comparisons of prognosis of MINOCA and MI-CAD patients is difficult due to the different pathophysiological mechanisms. In MINOCA the prognosis is closely related to the different and multiple causes of disease, which should be actively investigated [6]. The incidence of major cardiovascular events (MACE) in MINOCA patients has increased in the past few years [6].

It is important to remember that ischemic damage can be the consequence of a problem involving both the epicardial coronaries and the microcirculation. Following this pathophysiological concept, Scalone et al. [7], in a recent review, proposed a classification of the causes of MINOCA in epicardial and microvascular. The difference is given by the presence of ventricular motion abnormalities, identifiable on echocardiogram or ventriculography. In the case of epicardial vessels disease, there is a characteristic territoriality motion abnormality, compatible with the involved coronary artery; in the case of a dysfunction affecting the microcirculation, these abnormalities do not agree with the epicardial blood supply territory.

According to the underlying pathophysiological mechanisms, MINOCA are, therefore, differentiated between type I and type II MIs: type 1 MI caused by atherosclerotic plaque disruption, and type 2 MI due to non-atherothrombotic mechanisms (epicardial coronary vasospasm, coronary microvascular dysfunction, coronary thromboembolism, spontaneous coronary artery dissection, and supply–demand mismatch) [8].

The European Society of Cardiology (ESC) working group established a diagnostic protocol that include a complete medical history, physical examination, laboratory tests, imaging, and invasive procedures to uncover the underlying cause of MINOCA [1].

There are small number of studies which investigated the prognostic risk factor so far. A recent paper has shown that reduced left ventricular ejection fraction (LVEF), nonobstructive CAD, β-blockers during follow-up, and ST depression on ECG at admission are independent predictors of the long-term prognosis of MINOCA patients [9]. Research on Chinese MINOCA patients reported that older age, female sex, atrial fibrillation, and reduced LVEF are independent predictors of MACE [10].

A study based on data from the SWEDEHEART registry [11], observed that approximately 6.3% of MINOCA patients experienced a recurrence of infarction in an average period of 17 months. About half of the patients, had progression of coronary lesions on angiographic re-evaluation. Furthermore, the mortality rate was overall non-negligible, with no significant differences between the subgroup with a second event classifiable as MINOCA and the subgroup with obstructive coronary artery disease (13.9 vs. 11.9%, *p* = 0.54) [11]. In MINOCA patients the long-term quality of life also seems to be compromised: the persistence of anginal symptoms at 1 year was documented in 25% of the patients [12].

Early cardiac magnetic resonance (CMR) is a pivotal diagnostic tool that may provide a differential diagnosis for 60% to 80% of MINOCA cases according to several previous studies [13,14,15,16,17,18]: subendocardial late gadolinium enhancement (LGE) is the most frequent pattern consistent with infarct. Performing CMR within 7 days from clinical presentation is the optimal time to increase the diagnostic accuracy [19].

Intracoronary (IC) imaging such as optical coherence tomography (OCT) and at lesser extent intravascular ultrasound (IVUS) are important tools for the diagnosis of coronary plaque disruption, spontaneous coronary dissection, and coronary thrombosis.

This article aims to review the literature about the role of intracoronary imaging in the diagnosis of MINOCA.

## 2. Role of OCT

**Figure 1:**
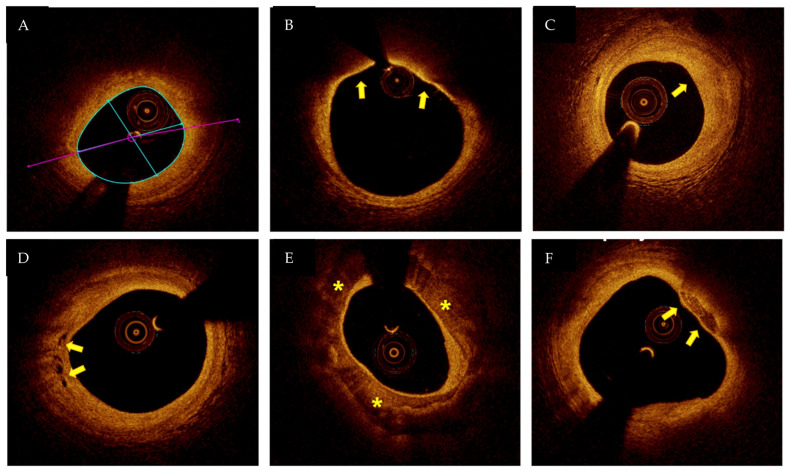
OCT (optical coherent tomography image): (**A**) lipid-rich plaque; (**B**) thin-cap fibroatheroma (arrows); (**C**) fibrous plaque (arrow); (**D**) neovascularization (arrows); (**E**) calcification (asterisks); (**F**) spotty calcium (arrow). Figure from authors’ library.

**Figure 2:**
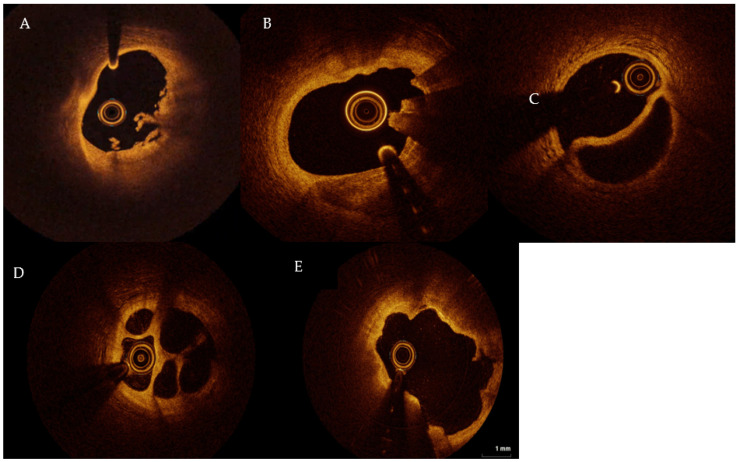
(**A**) Plaque erosion; (**B**) calcified nodule; (**C**) SCAD; (**D**) honeycomb-like structure, hinting recanalized thrombus; (**E**) plaque rupture. SCAD: spontaneous coronary artery dissection. TL: true lumen, FL: false lumen. Figures from authors’ library.

OCT (Abbott, USA) is a high-resolution method for plaque characterization [20]. The principle by which OCT works is similar to ultrasound but near-infrared light waves (in the 1300 nm range) are used instead of ultrasounds to generate high-resolution images (range 15–20 µm). The light waves, emitted into the vessel through a special catheter positioned in the coronary artery, meet the surrounding structures that are partly absorbed and partly reflected by them. OCT is able to distinguish the different components of the plaque (fibrous cap, thrombus, and calcification) exploiting the different optical properties of each tissue [18] (Figure 1). The different layers (intima, media, and adventitia) of the vessel wall can be accurately visualized by OCT [21]. Angiographic images may suggest plaque disruption, but information regarding the exact pathogenic mechanism responsible for MINOCA cannot be obtained from angiography alone [22,23,24,25,26] (Figure 2). The underlying thrombotic process may be accompanied by peripheral thromboembolism and/or vasospasm. This is the reason why a complete vascular occlusion may not be visible by the angiography alone.

Despite its potentiality, the role of OCT in the management of obstructive CAD is well-established [27], while the experience with this tool in the context of MINOCA is still limited. To date, a small number of observational studies showed that OCT is an accurate method to visualize plaque disruption or thrombi in MINOCA and to define the prognosis.

Yamamoto et al. (a single center retrospective study) [28] found that among patients presenting with ischemic symptoms and/or signs, but angiographically nonobstructive culprit lesions, approximately 25% had abnormal findings by OCT (namely thrombus, plaque rupture, calcified nodule, or intimal lacerations) whether patients presented with acute/unstable or stable coronary artery disease. Usui et al. [29] used OCT to identify differences between woman with MINOCA compared with woman with obstructive coronary lesion (MI-CAD). There was a higher prevalence of recent ruptured plaque, intraplaque hemorrhage in MINOCA patients. Plaque rupture with a persistent cavity and thrombus was, instead, more often found in MI-CAD. These findings may suggest a different etiopathogenetic mechanism underling MINOCA that could be highlighted by OCT.

Taruya et al. investigated the role of OCT in defining the prognosis of MINOCA [30]. Eighty-two patients with acute coronary syndrome (ACS), but non-obstructive CAD, were included. OCT revealed 42 hidden high-risk lesions (51.2%). During the follow up (2 years), 4 of the 42 (10%) patients with high-risk lesion experienced ACS, whose culprit lesion could be identified in the same segment where the index high-risk lesion was found.

A more recent observational study (Mas-Lladò et al.) [31] enrolled 10 patients in approximately 5 years who manifested exercise-related ASC in the absence of significant coronary artery stenosis. They found that eight patients had an atherosclerotic plaque, one1 patient had a spontaneous coronary dissection and in only one case there was no OCT findings.

Zeng et al. [32] studied 190 MINOCA patients using OCT(Abbott, USA) They found atherosclerotic causes of MINOCA (Ath-MINOCA) (plaque erosion, plaque rupture, and calcified nodule), in 64 patients (33,7%). The causes of non-atherosclerotic MINOCA occurred instead in 91 patients (47.9%) (spontaneous artery dissection and coronary spasm). Compared with patients with nonatherosclerotic mechanism, those with Ath-MINOCA had worse clinical outcomes with a higher incidence of major adverse cardiac events (MACE) (15.3% vs. 4.5%; *p* = 0.015), more frequent target lesions revascularization (TLRs) (6.1% vs. 0%; *p* 1⁄4 0.030) and more rehospitalizations for angina (6.1% vs. 0%; *p* = 0.030).

However, it should be noted that even in MINOCA due to coronary causes, OCT may not always provide a definite diagnosis. For example, in the presence of epicardial coronary spasm, OCT may only generate some hints. The characteristic that can be observed in spasm lesions by OCT is an intimal bumping at baseline and intimal gathering during spasm compared with the no spasm lesion [33]. The prevalence of coronary spasm is variable. It has been reported that about a quarter of patients with MINOCA have microvascular spasm [34]. A provocative test in this context is safe and identifies a subset of high-risk patients [35].

Spontaneous coronary artery dissection (SCAD) is an uncommon cause of MINOCA, more frequent in women than in men [36]. SCAD results in separation of the inner intimal lining from the outer vessel wall. The incidence is between 0.07% and 1.1%, but in many cases, could be missed or misdiagnosed [36]. Triggers for SCAD could be extreme physical exertion (particularly in young male patients), intense emotional stress, sympathomimetic drugs, childbirth, and Valsalva-like activities. These mechanisms increase shear stress on the coronary artery wall, by elevating catecholamine levels and intra-abdominal pressure [36]. Although long-term prognosis is excellent, the risk of recurrent SCAD events is significant, with an average rate of 5% per year [36]. A thorough examination, including clinical, and lesions associated risk factors may help to define the prognosis, the risk of recurrence, and drive a conservative vs. invasive management [37]. OCT can give essential information to define the precise length of intramural hematoma, the degree of luminal compromission, and the thickness of the dissected tear [38]. However, the procedural risk associated with OCT in the context of coronary dissections, due to the progression of the false lumen related to contrast injection, should be considered [39].

The disadvantages of OCT are related to the evaluation of proximal part of left main or right coronary artery or in case of coronary ectasia.

In synthesis, OCT is probably the highest resolution tool supporting the identification of different etiopathogenetic mechanisms underlying MINOCA. Further, according to recent evidence, although limited by the observational design, it may also have a pivotal relevance in defining the prognosis.

## 3. Role of IVUS

Intravascular ultrasound (IVUS) uses a special catheter, designed with a miniaturized ultrasound probe with a resolution of 100 μm. The proximal end of the catheter is attached to a computerized ultrasonic equipment. IVUS allows the application of ultrasonic technology (such as the piezoelectric transducer or the capacitive micromachined ultrasound transducer, CMUT) to see through the surrounding blood column, displaying the endothelium. Despite gray-scale IVUS-based atheromatous plaque, classification is limited due to its low spatial resolution classification in four categories has been suggested: 1 soft plaque, 2 fibrous plaque, 3 calcified plaque, 4 mixed plaques (no single acoustical subtype represents >80% of the plaques) [40]. Of note, enhancements of such ancillary technique currently used for research purposes such as the Near Infrared spectroscopy (NIRS)-IVUS may provide information about the lipid content in the arterial wall, detailing a component of plaque vulnerability. Since in MINOCA, culprit plaques with ulcerations tend to have large lipid components, it is likely that the clinical implementation of such technique will remarkably empower the range of tools helping to assess patients with MINOCA [41].

Compared to OCT, IVUS is a more widespread technique [42]. However, OCT offers a higher resolution and a more detailed evaluation at the endoluminal level [43].

On the other side, IVUS has some advantages: higher tissue penetration depth (4–8 mm, while OCT has 1–3 mm), therefore exploring the outer plaque layers, providing information on lipid content and vessel remodeling [44] is repeatable several times during the same procedure, and better visualizing calcified lesions which are conversely penetrated by OCT [45]. However, in OCT, reacquisition of images is also possible. Further, IVUS do not require contrast administration, therefore representing the tool of choice for patients suffering from chronic kidney disease. However, some studies valuated the image quality and diagnostic value of saline solution in OCT to be similar to contrast [46,47,48].

As for OCT, the role of IVUS in the management of obstructive CAD undergoing percutaneous revascularization is well-established [49]. In contrast, evidence evaluating the importance of IVUS in MINOCA is scant. However, when diagnostic uncertainty exists, IVUS is recommended to diagnose and to guide appropriate treatment.

Noguchi et al. [50], in a recent observational study, found that the plaque burden in non-obstructive left main coronary artery (LMCA) defined by IVUS, was independently associated with long-term all-cause and cardiac mortality in patients not undergoing LMCA revascularization, even when the lumen area was preserved.

**Figure 3:**
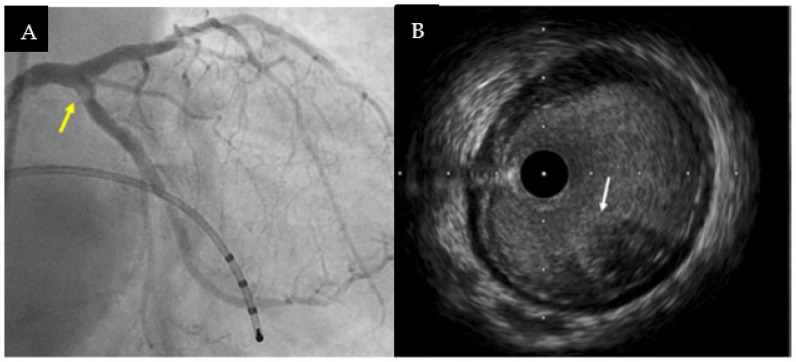
(**A**) Medical history suggesting a cardioembolic etiology for a patient admitted for inferior ST—segment elevation myocardial infarction with cardiac arrest on onset. Coronary angiography revealed haziness on ostial left circumflex artery (arrow). (**B**) IVUS highlighted intimal thickening with some backscattering to the lumen of the vessel, confirming the presence of intracoronary embolus without local atherosclerosis (arrow). The case was successfully managed with thrombus-aspiration and without stent implantation. Figures from authors’ library.

Some case reports hint that IVUS may be a game-changer in the management of patients presenting with ACS or cardiac arrest but without obstructive CAD (Figure 3) [51].

The reproducibility and availability of the IVUS means that it can be very useful when the mechanism of the MI refers to a microvascular disfunction.

### Hybrid IVUS–OCT Imaging

Hybrid imaging where complementary OCT and IVUS modalities are integrated, has been hypothesized to improve the plaque characterization. For example, the increased tissue penetration of IVUS allows identification of positive remodeling while the superior resolution of OCT permits measurement of fibrous cap thickness [52].

Hybrid IVUS-OCT imaging should act to reduce imaging artifacts, which are well characterized in both OCT and IVUS imaging datasets [53].

Hybrid IVUS–OCT imaging can be performed using separate catheters; such procedures may result in increased risk of complications. Significant effort has, therefore, been made to develop a novel combined IVUS–OCT catheter that can simultaneously generate images from the same arterial cross-sectional plane. The first-generation catheters were bulky and suffered from spatial mismatches, but the newest ones are <1.4 mm in diameter. These technological advances will facilitate the development of a combined IVUS–OCT platform, which will improve atherosclerotic plaque characterization [54].

## 4. Integrating Invasive and Non-Invasive Cardiac Imaging: The Role of CMR

European guidelines (class I, level of evidence B) [55]) and position papers highlighted the diagnostic potential of CMR in patients with suspected MINOCA [56].

CMR is a noninvasive technique that uses intrinsic tissue contrast to obtain three-dimensional functional and anatomical information on the heart [57].

When performed soon after MI, CMR enables a definitive diagnosis, and is useful for additional work-up, management and risk prediction [57]. CMR could define myocardial activity, tissue morphology, myocardial edema, myocardial perfusion, coronary resistance, and diastolic filling under the endocardium and pericardium.

CMR is the gold standard for volumetric and functional analyses of both ventricles; late gadolinium enhancement (LGE) is an important predictor of adverse events in ischemic and non-ischemic cardiomyopathies [58].

Acute changes in the myocardium after coronary occlusion reveal increased water content in tissues [57]. In the first 10 min following coronary occlusion, the lack of blood determines intracellular metabolic and molecular changes with accumulation of osmotically active substances within the myocytes [59]. Restricted blood flow due to upstream epicardial artery obstruction determines a pathophysiological cascade of progressive myocardial injury [57]. Ischemia lasting 20–30 min followed by reperfusion leads to myocardial edema. This observation introduced the possibility to detect by CMR acutely ischemic myocardium and to differentiate it from chronic myocardial infarction [60].

However, due to the poor availability of the methodic, the application of CMR in the acute setting is not widely used in clinical practice.

Myocardial perfusion, changes in myocardial tissue composition from early ischemia through to necrosis, and the consequent changes in myocardial function could be defined by different CMR complementary techniques [57]. Long-term ischemia leads to irreversible injury that progresses from the subendocardium towards the epicardium, and becomes transmural by 6–12 h. An intramyocardial or subepicardial LGE localization (non-ischemic pattern), recognizes other causes (e.g., myocarditis, cardiomyopathies, etc.)

Using the contrast agents, such as gadolinium diethylenetriaminepentaacetic acid (Gd-DTPA), microvascular dysfunction appears as a dark hypo enhanced core within the hyperenhanced infarcted area. The microvessels that are plugged with thrombus, cells, and edema are temporary inaccessibility of the infarct core to Gd-DTPA [61].

Owing to multiplanar and three-dimensional imaging capabilities, high tissue contrast, real-time imaging, absence of ionizing radiation, and the ability to track cells and monitor their migration, CMR techniques could become the best imaging option in assessing not only the diagnosis but also the prognosis of MI [62].

A small number of studies evaluated the diagnostic performance by OCT integrated by CMR (Table 1).

Gerbaud et al. [63] described 40 patients with MINOCA undergoing OCT and CMR. Acute myocardial infarction was evident at CMR in 31 of 40 patients (77.5%) and by coupling OCT with CMR, a substrate and/diagnosis was found in 100% of cases. OCT findings were frequently accompanied with corresponding myocardial injury confirmed by CMR. Plaque rupture and plaque erosion were observed in 35% and 30% of patients presenting with MINOCA, respectively.

Opolski et al. enrolled 38 MINOCA patients [64]. OCT found plaque disruption and intracoronary thrombus in 24% and 18% of patients, respectively. Among 31 patients undergoing CMR, an ischemic-type LGE was present in 21% and was more common in patients with plaque disruption (50% versus 13%, respectively; *p* = 0.053) and coronary thrombus (67% versus 12%, respectively; *p* = 0.014).

Reynolds et al. [65] conducted the largest study, including 145 women with MINOCA who underwent multivessel (ideally three-vessel) OCT followed by CMR.

A lesion visible on OCT could be identified in 42% of patients with CMR-detected infarction. An ischemic cause was identified in 63.8% of women, a nonischemic cause was identified in 20.7%, and no mechanism was identified in 15.5%.

Some years earlier, Reynolds et al. [66] enrolled 121 women with no obstructive CAD. They performed IVUS during angiography and CMR one week later. Plaque disruption was observed in 38% of patients undergoing IVUS. CMR demonstrated abnormalities in 26/44 patients (59%). Non ischemic pattern was also observed but the most common LGE pattern was ischemic. This study demonstrates that IVUS and CMR provide complementary mechanistic insights in MI patients.

When MINOCA in suspected, the CMR is essential in the diagnostic work up.

## 5. Diagnostic Approach

**Figure 4:**
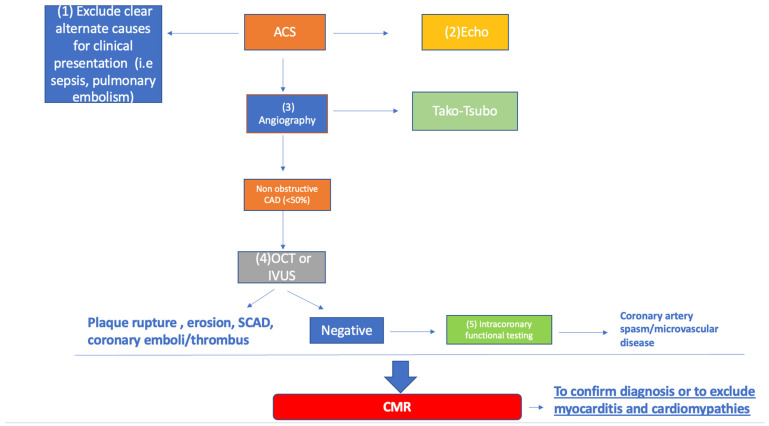
Proposed approach to MINOCA. Flowchart is explained in the text. ACS: acute coronary syndrome; CMR: cardiac magnetic resonance; MINOCA: myocardial infarction with non-obstructive coronary artery disease; OCT: optical coherence tomography; SCAD: spontaneous coronary artery dissection.

The necessity of intravascular imaging tailored to the patient’s characteristic in MINOCA setting is largely supported by the clinical need of pursuing a definite diagnosis, despite the evidence in this sense being scant. A proposed diagnostic approach by the authors in summarized in Figure 4.

In the contest of significant rise and fall of cardiac damage biomarkers mimicking an ACS, the first step is excluding alternative diagnosis (sepsis, pulmonary embolism, artery dissection, and myocarditis) with proper analysis of medical history, ECG, and trans thoracic echocardiography.

In the most recent guidelines [55], myocarditis has been excluded from diagnostic MINOCA’s panel. However, some evidence suggested that myocarditis sustained by parvovirus B19 recognizes a component of microcirculation dysfunction resulting in a vasoconstriction [67]. Coronary vasospasm is one of the main reasons for atypical chest pain in patients with biopsy-proven PVB19 myocarditis [67]. In these patients, subendocardial lesions may occur as a consequence of coronary vasospasm making the diagnosis challenging. The clinical presentation can often suggest various diagnoses and it is not always possible to identify a certain etiological cause.

During coronary angiography the diagnosis of MINOCA may be suspected.

Left-ventricle angiography, with the demonstration of the absence of significant stenoses, is able to document the classic alterations of ventricular motion that could identify or exclude Takotsubo cardiomyopathy. The other differential element is the reversible nature of the left ventricular dysfunction (usually 4–8 weeks). LGE is generally absent, although modest diffuse LGE may sometimes be present in segments with impaired motions [68].

At this point OCT or IVUS should be performed.

If there is the suspect of coronary artery spasm or microvascular disease, intracoronary functional testing such intracoronary acetylcholine provocation should be considered.

Coronary Vasomotion Disorders International Study Group (COVADIS), standardized the diagnostic criteria of vasospastic angina [68]. The main “red flags” that suggests this diagnosis concern the anamnestic presence of almost exclusively anginal episodes at rest or during the night, reduced by nitrates or calcium channel blockers, which can be exacerbated by hyperventilation and smoking [68].

Coronary angiography has a very low sensitivity for the identification vasospastic angina (VSA). Intracoronary infusion of vasoactive agents (acetylcholine, ergonovine, and substance p) allows for the assessment of coronary vascular function. This represents the gold standard for the diagnosis of VSA, with more than 90% sensitivity and 99% specificity [69]. If there is a change in ischemic ECG without an epicardial spasm, microvascular angina might be diagnosed.

The frequency of major complications in intracoronary infusion of vasoactive agents according to Ciliberti’s observational study ranges from 0% to 4.9%, while the rate of minor complications ranges from 0% to 16.3% [70]. The most common major complication is ventricular fibrillation (VF) or sustained ventricular tachycardia (SVT) occurring in 0.69% of the cases, while shock (0.03%), myocardial infarction (0.01%), and prolonged/refractory spasm (0.01%) are rare [65]. Safety concerns about intracoronary administration of ACh, still persist limiting its use in clinical practice.

Coronary thromboembolism may represent an overlapping mechanism of atheromatous plaque rupture or vasospasm, but may also be the only responsible for MINOCA.

Coronary thromboembolic episodes can be direct or paradoxical. Direct forms are characterized by the localization of the embolus source in the left sections of the heart: atrium, ventricle, pulmonary veins, endocardium processes involving the mitral and aortic valves (more rarely cardiac tumors), and also a primary origin in the coronary district (for example from embolization of more proximal coronary aneurysms) [71]. In the paradoxical forms, the genesis of the thrombus occurs in the venous circulation and, through the presence of an intracardiac communication (oval foramen or atrial septal defect), there is a subsequent passage in the coronary district [72].

In the diagnostic workup, all conditions potentially associated with the development of thromboembolism must be excluded: it is necessary to search for the presence of atrial fibrillation and endocardial processes, which represent, respectively, the first and second most frequent sources of embolism [73]. In a systematic review, a prevalence of 14% of hereditary thrombophilic disorders was found among patients with MINOCA [6]. Despite this, the role regarding the routine application of screening tests for hereditary thrombophilia in patients with suspected coronary thromboembolism is not clear.

Transesophageal and/or contrast-enhanced echocardiography can be used to detect the source of cardiac embolism in coronary microvascular embolization.

CMR is an excellent modality to discriminate between ischemic and non-ischemic mechanism of MINOCA, but cannot inform whether the ischemia was due to an atherothrombotic or non-atherothrombotic processes. LGE distribution, in addition to having a prognostic rule, provides guidance on possible alternative diagnoses such as myocarditis or cardiomyopathy.

In this context, the role of IC imaging appears paramount also to tailor patients with MINOCA to specific therapies.

Approximately 8–67% of MINOCA patients have a normal CMR finding, characterized by the absence of ventricular motion abnormalities, edema, or LGE [64]. This may depend on the time intercurrent between the execution of the exam and the acute event, the age of the population studied, the different percentage composition of male/female, the presence of modest diffuse fibrosis which is not identified with the standard sequences of LGE [74]. T1 and T2 mapping myocardial signal will certainly provide a contribution in improving the ability to identify pathological myocardial areas.

In our opinion, CMR should be performed as soon as possible.

When the CMR is normal, in absence of other clarifying diagnostic data, the definitive diagnosis remains impervious, and the treatment is empiric.

A recent meta-analysis from our group [75] suggested that beta-blockers, statins, and dual antiplatelet therapy (DAPT) are associated with a survival benefit among MINOCA patients, while ACE-inhibitors/Angiotensin receptor blockers reduce risk of MACE. However, due to the limited use of IC imaging within included studies, results are difficult to interpret. Indeed, it is likely that the benefit coming from DAPT therapy is restricted to patients with MINOCA due to plaque erosion/rupture. Patients with cardioembolic events may be more appropriately committed to anticoagulant therapies, whereas a vasospastic process may benefit from vasodilatory agents.

## 6. Conclusions

Assessing the impact of In this review, we summarized the role of intracoronary imaging in the diagnostic MINOCA work up. However, only observational studies supporting the use of IVUS and OCT exist.

IC imaging to drive a tailored therapy among MINOCA patients along with the cost-effectiveness ratio of such approach in a prospective study would be valuable.

Randomized trials evaluating the best medical therapy are currently missing.

## Figures and Tables

**Table 1:** OCT and CMR findings. SCAD: Spontaneous coronary artery dissection. MI: Myocardial infarction. CMR: Cardiovascular magnetic resonance. OCT: optical coherence tomography. Values are (%).

Name of the Study	*n* of Patients	Plaque Rupture	Plaque Erosion	In Situ Thrombosis	SCAD	MI at CMR
Gerbaud et al. [63]	40	14 (35)	12 (30)	3 (7.5)	2 (5)	31(77.5)
Opolski et al. [64]	38	9 (24)	NA	6 (16)	NA	8 (21)
Reynolds et al. [65]	145	8 (5.5)	NA	5 (3.4)		38 (26)

## Data Availability

Not applicable.
